# Do We Need to Breed for Regional Adaptation in Soybean?—Evaluation of Genotype-by-Location Interaction and Trait Stability of Soybean in Germany

**DOI:** 10.3390/plants12040756

**Published:** 2023-02-08

**Authors:** Cleo A. Döttinger, Volker Hahn, Willmar L. Leiser, Tobias Würschum

**Affiliations:** 1 Institute of Plant Breeding, Seed Science and Population Genetics, University of Hohenheim, 70599 Stuttgart, Germany; 2 State Plant Breeding Institute, University of Hohenheim, 70599 Stuttgart, Germany

**Keywords:** soybean, yield stability, genotype-by-environment interaction, genotype-by-location interaction

## Abstract

Soybean is a crop in high demand, in particular as a crucial source of plant protein. As a short-day plant, soybean is sensitive to the latitude of the growing site. Consequently, varieties that are well adapted to higher latitudes are required to expand the cultivation. In this study, we employed 50 soybean genotypes to perform a multi-location trial at seven locations across Germany in 2021. Two environmental target regions were determined following the latitude of the locations. Adaptation and trait stability of seed yield and protein content across all locations were evaluated using Genotype plus Genotype-by-Environment (GGE) biplots and Shukla’s stability variance. We found a moderate level of crossing-over type genotype-by-location interaction across all locations. Within the environmental target regions, the genotype-by-location interaction could be minimised. Despite the positive correlation (*R* = 0.59) of seed yield between the environmental target regions and the same best-performing genotype, the genotype rankings differed in part substantially. In conclusion, we found that soybean can be grown at a wide range of latitudes across Germany. However, the performance of genotypes differed between the northern and southern locations, with an 18.8% higher mean yield in the south. This in combination with the observed rank changes of high-performing genotypes between both environmental target regions suggests that selection targeted towards environments in northern Germany could improve soybean breeding for those higher latitude regions.

## 1. Introduction

Soybean (*Glycine max* (L.) Merr.) is a leguminous crop of globally high economic importance. Due to its high protein content, it is mainly used as animal feed. However, soybean products, such as tofu and dairy alternatives, are also very popular for human consumption with a steadily increasing demand in Germany [1,2] and worldwide [3]. In addition, soybean is also grown for its high oil content. As a crop that was domesticated in temperate China [4], soybean production requires warmer temperatures [5]. The largest producers are the USA, Brazil, and Argentina [6]. As a result of selection and breeding efforts, cultivars that mature earlier and are more tolerant to cooler temperatures are available nowadays [7,8]. This enables the successful cultivation of soybean at higher latitudes, like in Germany, despite the shorter growth periods and the generally lower temperatures, especially in spring. In Germany, the majority of the soybean acreage lies in the southern half and to date, there is only very little soybean cultivation in the more northern regions [9]. The southern regions are generally better suited for soybean cultivation due to temperature, rainfall patterns, solar radiation, and shorter photoperiod [5]. Photoperiod is a decisive factor in soybean cultivation since soybean is originally a short-day plant and as such requires short daylengths to induce flowering [10]. However, due to breeding efforts targeting this response, there are cultivars available that are less sensitive to long-day conditions, enabling soybean production at higher latitudes [11].

Germany has a high demand for soybean that is mainly covered by imports. Only around 2% of the amount of soybean imported into Germany is produced by German farmers [12]. In exporting countries, extensive soybean production leads to environmental issues such as the deforestation of rainforests [13]. In the context of the “Protein Plant Strategy”, with the aim to increase sustainable plant-based protein production and decrease the dependence on imports, the German government has promoted the expansion of the production of soybean and other legumes [14]. To increase the acreage and yield of soybean grown in Germany and to expand the cultivation across the whole country, especially to regions at higher latitudes, further breeding efforts are necessary. To successfully develop cultivars suitable for the different regions, a thorough understanding of the patterns of genotype-by-environment interaction is crucial.

Genotype-by-environment interaction describes the phenomenon of different genotypes responding differently to different environments, meaning the relative performance of the genotypes changes between environments. More specifically, crossover interaction leads to rank changes of genotypes between different environments. In order to deal with genotype-by-environment interaction, trait stability can aid in assessing a genotype’s suitability across a number of environments. Trait stability can be estimated following the static or dynamic concept [15,16]. Static stability describes the ability of a genotype to show the same absolute level of performance regardless of the environment. A dynamically stable genotype, by contrast, shows the same level of performance across environments but always relative to the environmental mean. In consequence, the absence of genotype-by-environment interaction leads to dynamic stability [17].

Two approaches to managing genotype-by-environment interaction in a breeding program are to either avoid it by selecting genotypes that show stable and high performance across environments (wide adaptation) [17] or to exploit it by selecting different genotypes for different mega environments (specific adaptation), which are environmentally similar subsets of environments. Since high stability is generally associated with low levels of genotype-by-environment interaction [17,18,19], it can be challenging to breed for wide adaptation. Consequently, when aiming to breed for wide adaptation, low levels of genotype-by-environment interaction or at least non-crossover interaction are required [20].

Genotype-by-environment interaction, or when analysed within a single year also referred to as genotype-by-location interaction, is a very common phenomenon for quantitative traits such as yield [21], but little is known for soybean in high latitude regions such as Germany in Central Europe. The objectives of this study were therefore to (i) evaluate the suitability of different regions in Germany for soybean production, (ii) investigate the magnitude and pattern of genotype-by-location interaction for soybean cultivated in Germany, and (iii) draw conclusions for soybean breeding in high latitude regions.

## 2. Results

**Figure 1:**
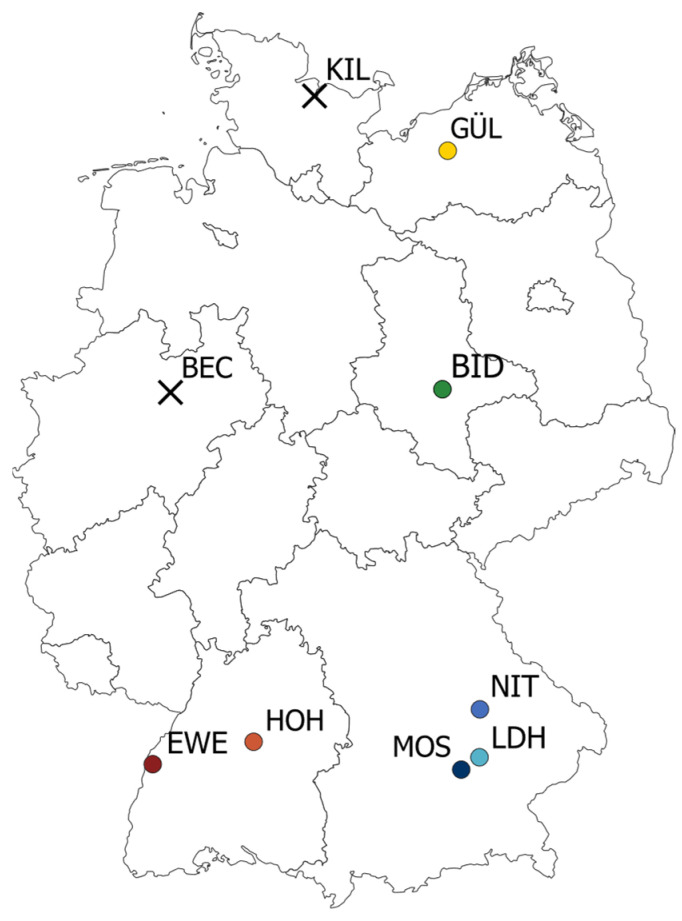
Map of test locations. Crosses show locations that could not be harvested.

This study analyses single-year data from seven locations with a focus on soybean performance in different regions in Germany. The locations in our study (Figure 1, Table 1, Figure S1) were separated into two environmental target regions based on their latitude, as this represents an important factor of soybean adaptation. Furthermore, this approach separates locations in the south, where soybean is already established, from the locations at higher latitudes, where there is barely any soybean cultivation. Therefore, the environmental target region in the southern half of Germany (STH) comprised the locations Eckartsweier (EWE), Hohenheim (HOH), Moosburg an der Isar (MOS), Niedertraubling (NIT), and Landshut (LDH). The environmental target region of the northern half of Germany (NTH) was assigned to the locations Biendorf (BID) and Gülzow-Prüzen (GÜL).

Across all seven locations, the mean seed yield was 30.37 dt ha^−1^ (Table 2) and across locations within the northern and southern half of Germany, the mean seed yield was 26.83 dt ha^−1^ and 31.82 dt ha^−1^, respectively. Within single locations, mean seed yield ranged from 24.26 dt ha^−1^ for BID to 36.57 dt ha^−1^ for EWE. Protein content was high with an average of 45.09% across all locations. In single locations mean protein content ranged from 42.87% in MOS to 47.29% in EWE. Note that data on protein content was not available for GÜL. Seed yield was significantly (*p* < 0.05) positively correlated between all locations except for GÜL and MOS (Figure S2). For protein content, all locations were highly significantly (*p* < 0.001) positively correlated (Figure S3). Most trait correlations were not significant (Figure S4). Exceptions were protein and oil content that were significantly (*p* < 0.001) negatively correlated (*R* = −0.91). Oil content was significantly (*p* < 0.05) positively correlated with seed yield at a moderate level (*R* = 0.31) and consequently protein content and seed yield were moderately negatively correlated (*R* = −0.20), though this was not significant. In addition, protein content was significantly (*p* < 0.05) positively correlated with days to maturity (*R* = 0.43), indicating that later maturing genotypes tended to have a higher protein content.

The genotypic variance was significant for all traits across locations, within environmental target regions STH and NTH, as well as within single locations (Table 2). In all cases was the genetic variance larger than the genotype-by-location interaction variance. The location variance was larger than the genotypic variance across all locations for all traits and within NTH for seed yield. Heritability across all locations was high to very high for all traits, ranging from 0.79 for plant height to 0.90 for seed yield and 0.94 for protein content (Table 2). In addition, heritability was very high for seed yield in STH (0.91), but in NTH was substantially lower at 0.55. For seed yield, repeatability in single locations ranged from moderate in GÜL and BID (0.53 and 0.54) to very high in NIT and MOS (0.88 and 0.92). For protein content, repeatability within single locations ranged from moderate in BID (0.55) to very high in EWE and NIT (0.94).

**Figure 2:**
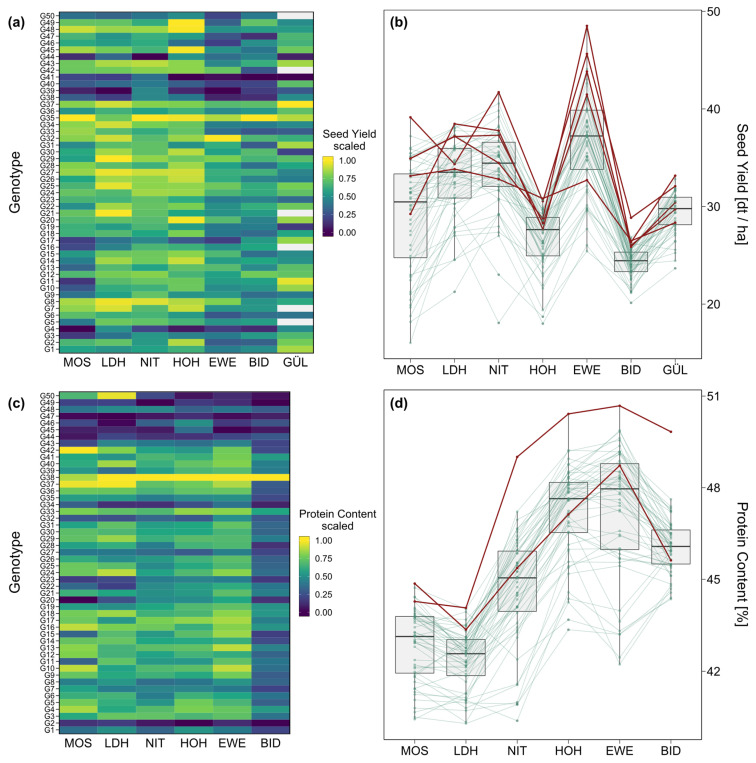
Heatmaps of (**a**) mean seed yield and (**c**) mean protein content for each genotype scaled within each environment to visualise the relative performance of the genotypes between environments. Boxplots of (**b**) seed yield and (**d**) protein content at each location. Red dots and lines show genotypes with the highest performance in at least one location. Green dots and lines show the performance of the remaining genotypes and the differences between locations.

In Figure 2a,c, the scaled seed yield and protein content for each genotype within each location are shown. For example, genotype G35 showed a high seed yield relative to the other genotypes within all locations and G38 showed very high protein content relative to the other genotypes in all locations. Figure 2b,d show boxplots of the best linear unbiased predictors (BLUP) within each location. The red lines indicate that for both seed yield and protein content, the genotypes performing best in at least one location had higher values than average in most locations. However, clear crossing-over interaction for seed yield, even for the highest-performing genotypes, could be observed (Figure 2b).

**Figure 3:**
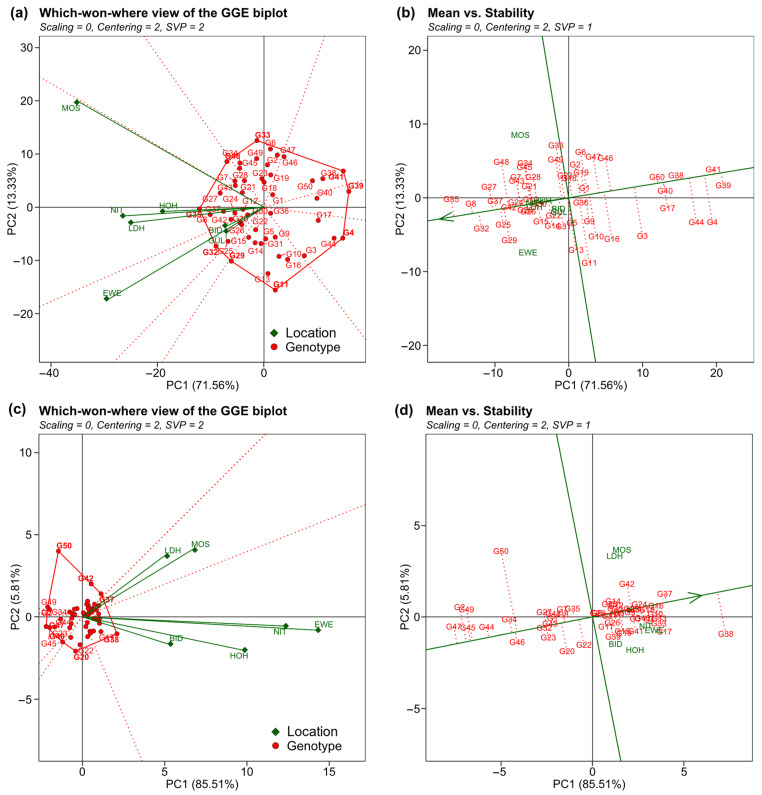
Location-centered GGE biplots for (**a**,**b**) seed yield and (**c**,**d**) protein content. Sections between red dotted lines include locations with the same highest-performing genotype (**a**,**c**), which is shown in bold in the respective section. Angles between location vectors indicate correlations between locations, where smaller angles represent a stronger positive correlation. Red dotted lines in mean vs stability plots (**b**,**d**) indicate stability across locations, where longer lines represent lower stability. The position of the genotypes on the green line represents the relative mean performance and the arrow points in the direction of higher performance.

**Figure 4:**
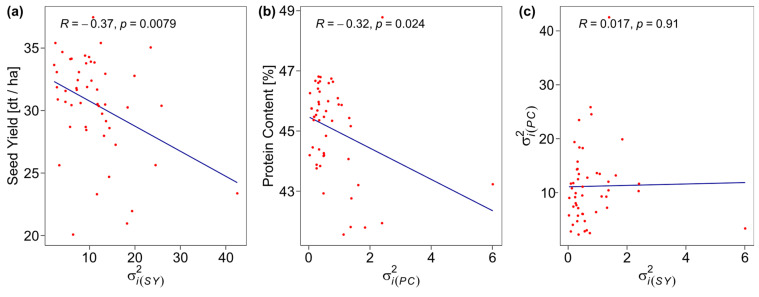
Correlation between (**a**) seed yield and the corresponding Shukla’s stability variance ($\sigma_{i\left(SY\right)}^{2}$), (**b**) protein content and the corresponding Shukla’s stability variance ($\sigma_{i\left(PC\right)}^{2}$), and (**c**) Shukla’s stability variances for seed yield ($\sigma_{i\left(SY\right)}^{2}$) and protein content ($\sigma_{i\left(PC\right)}^{2}$). Red dots show the values for each genotype, blue lines show the corresponding linear regression lines.

The angles between vectors in the Genotype plus Genotype-by-Environment (GGE) biplot for seed yield indicate a positive correlation between HOH, NIT, and LDH and between BID, GÜL, and EWE (Figure 3a). The performance vs. stability biplot (Figure 3b) indicates that G35 had the highest seed yield across locations while being mostly stable (relatively short red dotted line), whereas G39 was the lowest yielding genotype but had similar stability. Shukla’s stability variance ranged from 2.27 for G24 to 42.48 for G44 for seed yield (Table S1). As seed yield and stability variance for seed yield were significantly (*p* < 0.05) negatively correlated (*R* = −0.37) (Figure 4a), rankings between both measures differed.

**Figure 5:**
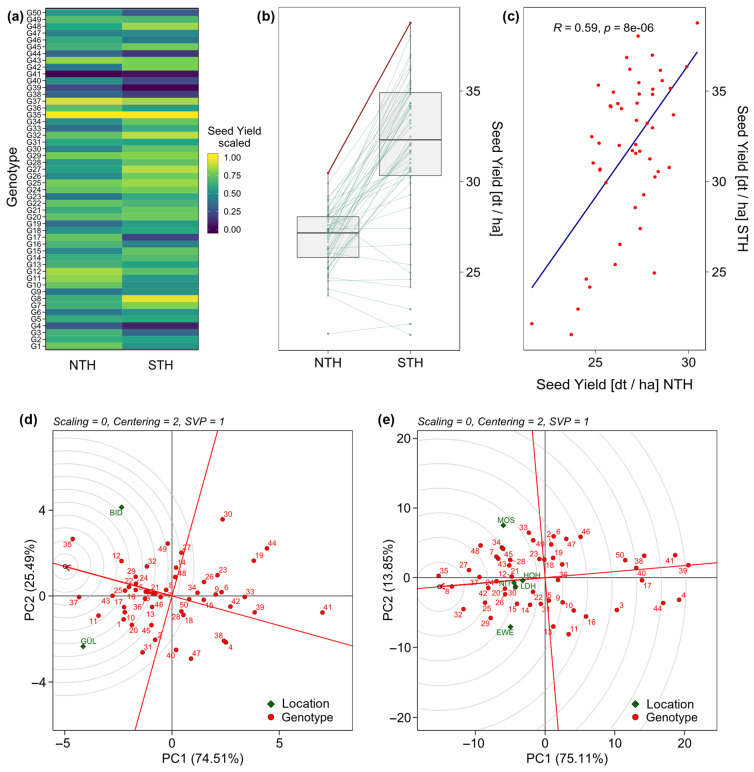
(**a**) Heatmap of mean seed yield scaled within each environmental target region, north (NTH) and south (STH), to visualise the relative performance of each genotype between environmental target regions. (**b**) Boxplot of mean seed in each target region. Red dots and lines show the highest-performing genotype in both regions. Green dots and lines show the remaining genotypes and their differences in performance between NTH and STH. (**c**) Correlation between mean seed yields in NTH and STH. Red dots show the values for each genotype. The blue line shows the corresponding linear regression line. GGE Biplots with singular value partitioning towards the genotypes ranking genotypes (**d**) in NTH and (**e**) STH based on both performance and stability. The red arrow points towards higher performance. A larger distance of a genotype to the respective red line indicates lower stability. The small circle at the tip of the arrow represents a fictional ideal genotype for the specific set of genotypes and locations. The further out of the center of the rings, the less ideal the genotype for the observed set of locations.

For protein content, the vector angles in the GGE biplot indicate positive correlations between all locations (Figure 3c). The respective performance vs. stability biplot (Figure 3d) shows G38 as the highest-performing but unstable genotype, and G47 as lowest performing while being relatively stable for protein content. The stability variance for protein content ranged from 0.03 for G43 to 6.01 for G50 (Table S2). Protein content and stability variance for protein content were significantly (*p* < 0.05) negatively correlated (*R* = −0.32) (Figure 4b). As observed for the means for seed yield and protein content (Figure S4), also their stability variances were not significantly correlated (Figure 4c). In NTH, seed yield ranged from 21.6 dt ha^−1^ to 30.47 dt ha^−1^, whereas in STH seed yield was generally higher, ranging from 21.54 dt ha^−1^ to 38.77 dt ha^−1^. Seed yield between NTH and STH was significantly (*p* < 0.05) positively correlated at *R* = 0.59 (Figure 5c). Further, the top performing genotype (G35) was the same in both NTH and STH (Figure 5a,b, Table 3), but for the other genotypes, some moderate crossing-over can be observed in the respective boxplot (Figure 5b). In addition, stability rankings according to Shukla’s stability variance were different between both environmental target regions. Stability variance for NTH ranged from 0 for G3, G8, G15, G24, G25, and G46 as the most stable genotypes to 83.21 for G44 as the least stable genotype. For STH, stability variance ranged from 0.40 for G40 as the most stable and 38.57 for G44 as the least stable genotype (Table 3). However, stability analysis for five genotypes (G5, G7, G16, G21, G42, and G50) was not possible due to missing observations in GÜL. GGE biplots, ranking genotypes based on both their performance and stability within both environmental target regions compared to a computed fictional ideal genotype, show differences in the most suitable genotypes for NTH and STH, except for G35, which emerged as most suitable for both (Figure 5d,e).

## 3. Discussion

### 3.1. Phenotypic Performance and Variance Components

Very high heritabilities across locations for both seed yield (0.90) and protein content (0.94) in conjunction with a lower, however highly significant (*p* < 0.001), genotype-by-location interaction variance component ($\sigma_{GL}^{2}$), illustrate a moderate level of genotype-by-location interaction (Table 2). For both traits, genotype-by-location interaction of the crossing-over type was observed, indicating the benefit of forming environmental target regions as subgroups of locations (Figure 2b,d). Importantly, to evaluate the repeatability of the observed interaction patterns, further evaluation across several years is required. The heritability for seed yield in STH was very high, but for NTH was substantially lower at 0.55. Accordingly, the two northern locations GÜL and BID forming the environmental target region NTH both showed lower repeatabilities (0.54 and 0.53), indicating a lower data quality in comparison to the other locations. This could be due to more inhomogeneous field conditions that could not sufficiently be accounted for by the field design or adaptation issues of at least some of the genotypes.

### 3.2. Performance and Trait Stability of Genotypes

In practical breeding, combining high performance and trait stability is a key objective to ensure reliable yields across different locations and years. In the case of this study, evaluating single-year data, yield stability via Shukla’s stability variance is analysed by estimating a genotype-specific $\sigma_{GL}^{2}$. Genotypes with stability variance $\sigma_{i}^{2}=0$ do not show any genotype-by-location interaction and are therefore considered perfectly stable across locations.

The top-performing genotypes for seed yield differed between locations showing a crossing-over between most locations (Figure 2b). In accordance with this, stability rankings were quite different from the performance rankings of the genotypes for seed yield across all locations (Table S1). However, the correlation between stability variance and seed yield was significant (*p* < 0.05) and negative at a moderate level (*R* = −0.34) (Figure 4a). This indicates a trend of higher-yielding genotypes tending to show higher yield stability across locations. However, the highest-yielding genotype G35 only reached the rank of 28 out of 50 in stability. Genotype G37, by contrast, ranked third in mean seed yield and second in stability, resulting in G37 being the closest to an ideal genotype for seed yield in our study. It should be noted that the rankings by performance were not identical to the respective GGE biplot (Figure 3b) but showed minor rank changes. This is likely due to the different methods of the *metan* package [22] to estimate the mean performance of each genotype.

Similar to the results for seed yield, protein content and its stability variance were moderately negatively correlated at *R* = −0.31 (Figure 4b). Stability variances for both traits were not significantly correlated with each other (Figure 4c). For protein content, only two top-performing genotypes were found, G38 in all locations but MOS, where G42 had the highest protein content (Figure 2c,d). Stability analysis according to Shukla’s stability variance, however, classified G38 as highly unstable with the rank 49 out of 50. Following the concept of dynamic stability, stability variance measures how the genotype performance changes relative to the environmental mean (Equation (1)). Genotype G38 did not follow this pattern. In consequence, despite being the superior genotype in almost all locations and in addition to showing high performance in MOS, G38 is not classified as stable. Notably, genotypes that vary around the location means will be classified as unstable, but also a genotype that performs better than the mean at every location. So from a practical point of view, classifying G38 as unstable is misleading since the main interest of a breeder lies in the consistently high performance of a genotype. Accordingly, it should be noted that trait performance (especially yield) is the most important breeding objective while stability measures can only support selection decisions. Notably, if the number of environments in a multi-environment trial is large enough, the estimation of mean performance across environments will automatically penalise unstable genotypes that vary around the environment means [23]. It should be noted here, that this study focuses on the genotypic stability across locations in one test year and therefore cannot provide conclusions about the trait stability across years.

### 3.3. Expansion of Soybean Cultivation to Higher Latitudes Is Possible

The mean seed yield across locations within STH was 18.6% higher than across locations within NTH (Table 2). However, the significant (*p* < 0.05) positive correlation (*R* = 0.59) between seed yield in NTH and STH (Figure 5c) suggests that genotypes with high performance in STH tended to also show high seed yield relative to the other genotypes in NTH. This is further highlighted and supported by the genotype mean comparison shown in Figure 5b. Despite the rank changes due to crossing-over genotype-by-location interaction between NTH and STH, high-ranking genotypes in STH tended to also rank higher in NTH in many cases. Generally, crossing-over type genotype-by-environment interaction can be met by different selection decisions between target regions or mega environments [17,23,24]. However, within mega environments or target regions, genotype-by-environment interaction should be minimised. In our case, this goal was reached with the environmental target regions, as the ratio of genotype-by-location interaction to genotypic variance ($\sigma_{GL}^{2}:\sigma_{G}^{2}$) decreased from 0.40 across all locations to 0.28 within NTH and 0.22 within STH.

When evaluating genotypes in both environmental target regions for high seed yield and yield stability in the year 2021, it appears that different selection decisions should be taken for NTH and STH (Figure 5d,e). The highest-performing genotype (G35) was the same in both target regions. However, for example, G8 was just as suitable for STH as G35, while it merely showed seed yield close to the mean in NTH. Consequently, despite the single-year analysis, the results indicate that selection decisions made for one environmental target region cannot confidently be transferred directly to another. To further explore this, we compared the mean seed yield of the five highest-ranking genotypes across all locations to the mean of the five highest-ranking genotypes within each of the two regions. Selecting for specific adaptation led to a 0.40% yield increase in STH compared to selection across all locations. For NTH, by contrast, the yield increase was higher at 2.32%. This analysis must, however, be treated with caution, due to the higher number of locations in STH. This is also an issue when analysing mean seed yield and stability across all seven locations (Figure 3b). Rankings and stability depictions are similar to those observed for STH. As an environmental target region consisting of five locations compared to NTH with only two locations, STH is overrepresented in the analysis across all locations. Keeping this in mind, our results indicate that separate analysis of environmental target regions enables more accurate selection against genotype-by-location interaction. However, short environment vectors for GÜL and BID (Figure 3a), proportional to the smaller variation within those locations (Figure 2b), show that both locations were not discriminating. This means that GÜL and BID were less suitable as test locations for the specific set of genotypes in this study compared to the other five locations [25,26]. One possible explanation could be less favourable environmental conditions during the growth period (Table 1), as especially in GÜL many genotypes did not fully mature before the field had to be harvested. However, we could not observe any single environmental factor explaining the substantially lower yields in NTH (Table 1). In consequence, the lower yields likely resulted from a combination of environmental conditions, including latitude.

The cause of genotype-by-environment interaction and trait instability are different sensitivities to environmental factors. In soybean, yield changes are often due to differences in tolerances to cooler temperatures [27,28,29] and photoperiod [11,30]. The aim of the differentiation between NTH and STH as environmental target regions was to mainly capture the effect of different latitudes on seed yield. In the north of Germany, due to typically lower temperatures and longer day length, earlier maturing genotypes are required compared to southern Germany in order to maximise the yield potential [29,30,31]. This generally indicates the need for selecting different genotypes for different latitudinal regions, which warrants further research. The majority of the genotypes evaluated in this study stem from a breeding program developed in the south of Germany at the location of EWE. Consequently, they were previously selected for high performance in that region. A question of practical relevance is therefore, whether we can successfully select soybeans in the south for cultivation in the north of Germany. Despite the correlation between the two regions, we found that evaluations made in the south did not sufficiently reflect the performance at higher latitudes. Consequently, specific selection decisions made separately in each of those regions appear necessary in soybean breeding. Nevertheless, the trend highlighted in this study needs to be validated by further research, also taking the effect of different growing seasons into account. Further, in practice, selection within mega environments or environmental target regions is only appropriate if the response to selection can be substantially increased [24]. Alternatively, an in part shared selection, for example, for simple traits in early generations, may be recommended.

### 3.4. Conclusions for Soybean Breeding at Higher Latitudes

This study illustrates that soybean can be cultivated in different regions in Germany, covering a wide range of latitudes from south to north. Notably, however, the suitability of genotypes for cultivation in southern Germany and more northern regions differed in part substantially. Consequently, the trend observed for the year 2021 indicates that different selection in breeding programs specifically targeting different regions appears beneficial. Key environmental influences affecting performance and therefore also genotype-by-environment interaction and stability in soybean are latitude, temperature, and precipitation [27,28,29,30,31,32]. Notably, to reliably classify mega environments, knowledge about the stability of the genotype-by-location interaction patterns over several years is necessary [17]. Patterns that are not repeatable across years cannot be utilised [23] and are therefore of no use for specific cultivar selection. Therefore, further research evaluating multi-environment trials comprising several years is crucial for a deeper understanding of these trends. Consequently, in addition to further exploring the environmental target regions defined in this study, it would be interesting to investigate the patterns of genotype-by-location interaction more thoroughly by evaluating a larger number of locations in different regions covering a wide range of latitudes across several years. An unconventional but promising approach to do so is citizen science which allows for a dense coverage of locations across an entire country or even across countries [33]. This would also offer further insights into the significance of latitude and specific weather conditions in the formation of environmental target regions and mega environments relevant to soybean breeding at higher latitudes.

## 4. Materials and Methods

### 4.1. Plant Material and Field Trials

We evaluated a total of 50 genotypes with favourable tofu quality, including eight registered cultivars (Table S3). The field trials were conducted in 2021 at seven locations in Germany: EWE, HOH, MOS, NIT, LDH, BID, and GÜL (Figure 1, Table 1). Further two trial locations in Beckum (BEC) and Kiel (KIL) could not provide data, as at these locations the soybeans did not reach maturity due to bird pests and unfavourable weather conditions throughout the year. Weather data for the locations MOS and NIT were obtained from Agrarmeteorologie Bayern [34]. Weather data for the locations in Baden-Württemberg (EWE and HOH) were obtained from Agrarmeteorologie Baden-Württemberg [35]. For LDH, BID, and GÜL weather data were obtained from the Climate Data Center [36].

The trials were designed in an alpha lattice design with two replications, ten blocks per replication, and five genotypes per block. The soybeans were sown in four rows in plots of 6 m length and 1.5 m width. Standard agricultural practices for soybean production were followed. Seed yield in dt ha^−1^ was measured on the combine harvester and corrected for seed moisture content. Protein and oil content in percent were measured on dried seed (9–10% moisture content) in the laboratory with near-infrared spectroscopy (NIRS) using a Polytec PSS 2120 diode spectrometer and the corresponding software PSS-HOP (Polytec GmbH, Waldenbronn, Germany). Days to maturity were assessed as days between sowing and maturity determined by dry pods rustling when touched (growth stage R8). Further, plant height in cm and kernel dry matter in percent were measured.

### 4.2. Phenotypic Analysis

BLUPs and variance components were calculated across all locations and across locations within environmental target regions with the model

$$y_{ijkm}=\mu +g_{i}+l_{j}+gl_{ij}+r_{jk}+b_{jkm}+\varepsilon_{ijkm}$$

where *y_ijkm_* describes the phenotypic observation, *µ* is the intercept, *g_i_* is the effect of genotype *i*, *l_j_* is the effect of location *j*, *gl_ij_* is the interaction between genotype *i* and location *j*, *r_jk_* is the effect of replication *k* within location *j*, *b_jkm_* is the effect of block *m* within replication *k* in location *j*, and *ε_ijkm_* is the corresponding residual error term. BLUPs and variance components within locations were calculated with the model

$$y_{ijk}=\mu +g_{i}+r_{j}+b_{jk}+\varepsilon_{ijk}$$

where *y_ijk_* describes the phenotypic observation, *µ* is the intercept, *g_i_* is the effect of genotype *i*, *r_j_* is the effect of replication *j*, *b_jk_* is the effect of block *k* within replication *j*, and *ε_ijk_* is the corresponding residual error term. For both models, the variance components were tested for significance using likelihood ratio tests. Heritability (*h*²) and repeatability (*w*²) were calculated as proposed by Cullis et al. [37] and described by Piepho and Möhring [38]:

$$\bar{h^{2}}=1-\frac{\bar{\vartheta_{BLUP}}}{2\sigma_{g}^{2}}\;or\;\bar{w^{2}}=1-\frac{\bar{\vartheta_{BLUP}}}{2\sigma_{g}^{2}}$$

where $\bar{\vartheta_{BLUP}}$ describes the mean variance of the difference between two BLUPs and $\sigma_{g}^{2}$ is the genotypic variance. The sole difference between these values is the calculation across environments (*h*²) and the calculation within environments (*w*²). Standardised values were calculated following the min-max normalisation. Correlations were calculated as Pearson’s product-moment correlation between pairwise complete observations.

### 4.3. Genotype-by-Location Interaction and Stability Analysis

Environmental target regions were defined by their latitude as an important component of soybean adaptation. For this, locations were separated into two groups by their geographic location: GÜL and BID in the northern half of Germany (NTH) and EWE, HOH, MOS, NIT, and LDH with rather similar latitudes in the south of Germany (STH).

Genotype plus Genotype-by-Environment (GGE) biplot is an approach proposed by Yan et al. [39]. It aids the interpretation of genotype-by-environment interaction by visualising genotype-by-environment interaction patterns in the form of a principal component analysis (PCA) biplot [26,39]. GGE biplot can be used to investigate the relationships between environments and the differences between genotypes across environments [25,26]. Further, different GGE biplots can be used to visualise the stability of genotypes and aid in selection decisions by combining performance and stability parameters within a single plot.

Genotype-by-location interaction was analysed using GGE biplot analysis [25,26] based on the equation

$$y_{ij}-\mu -\beta_{j}=\lambda_{1}\xi_{i1}\eta_{1j}+\lambda_{2}\xi_{i2}\eta_{2j}+\varepsilon_{ij}$$

where *y_ij_* is the mean performance of genotype *i* in location *j*, *λ*_1_, and *λ*_2_ are the singular values, *ξ_i_*_1_, and *ξ_i_*_2_ are the eigenvectors of genotype *i*, and *η*_1*j*_ and *η*_2*j*_ are the eigenvectors of location *j* for the two largest principal components (PC1 and PC2), respectively, and *ε_ij_* is the corresponding error term. All plots were made without scaling and using environment-centering. To evaluate the relationships between locations, singular values were partitioned into the locations (SVP = 2). In order to compare genotypes, singular values were partitioned completely into the genotypes (SVP = 1).

Dynamic stability of the genotypes across locations was measured by estimating Shukla’s stability variance ($\sigma_{i}^{2}$) [40] based on the equation

(1) $$\hat{\sigma_{i}^{2}}=\frac{1}{\left(G-1\right)\left(G-2\right)\left(E-1\right)}[G\left(G-1\right)\underset{j}{\sum }{\left(y_{ij}-\bar{y_{.j}}-\bar{y_{i.}}+\bar{y_{..}}\right)}^{2}-\underset{i}{\sum }\underset{j}{\sum }{\left(y_{ij}-\bar{y_{.j}}-\bar{y_{i.}}+\bar{y_{..}}\right)}^{2}]$$

where *G* is the number of genotypes, *E* is the number of locations, and *y_ij_* is the mean performance of genotype *i* in location *j*. Negative estimated values for $\sigma_{i}^{2}$ were set to 0 [40].

All analyses were conducted with R within the *RStudio* [41] environment. BLUPs and variance components were calculated using the package *ASReml-R* [42]. GGE biplots, genotype-by-location heatmaps, and stability were calculated and produced using the package *metan* [22]. All graphs were drawn with the package *ggplot2* [43]. Correlation matrices were produced with the extension package *ggcorrplot2* [44].

## Figures and Tables

**Table 1:** Environmental characterization of test locations, including latitude (LAT), altitude above sea level (ALT), mean temperature (TEMP), and precipitation sum (PCPN) across the growth period and for each month within the growth period, soil group (SG), soil type (ST), and sowing date (SD).

		EWE	HOH	MOS	NIT	LDH	BID	GÜL
LAT		N 48°31′ 17.1876	N 48°43′ 18.048	N 48°26′ 35.2536	N 48°57′ 35.4564	N 48°32′ 38.4252	N 51°45′ 7.1568	N 53°49′ 12.504
ALT		141 m	400 m	440 m	334 m	403 m	80 m	14 m
TEMP	Apr–Oct	14.96 °C	13.63 °C	14.02 °C	14.04 °C	13.82 °C	14.74 °C	14.19 °C
	Apr	8.19 °C	6.95 °C	7.05 °C	7.11 °C	6.87 °C	6.73 °C	6.21 °C
	May	12.17 °C	10.77 °C	11.75 °C	11.29 °C	10.75 °C	12.13 °C	11.00 °C
	Jun	20.55 °C	19.22 °C	19.72 °C	19.86 °C	19.53 °C	20.21 °C	19.65 °C
	Jul	19.34 °C	18.13 °C	18.97 °C	19.00 °C	18.45 °C	19.74 °C	19.64 °C
	Aug	18.10 °C	16.51 °C	17.17 °C	17.14 °C	16.75 °C	17.59 °C	16.72 °C
	Sep	16.33 °C	15.10 °C	15.20 °C	15.50 °C	15.86 °C	16.29 °C	15.42 °C
	Oct	10.05 °C	8.75 °C	8.26 °C	8.38 °C	8.56 °C	10.50 °C	10.67 °C
PCPN	Apr–Oct	481.8 mm	432.5 mm	821.7 mm	424.5 mm	724.2 mm	373.8 mm	460.9 mm
	Apr	44.8 mm	36.2 mm	51.0 mm	14.1 mm	21.1 mm	23.7 mm	41.8 mm
	May	114.4 mm	72.0 mm	97.6 mm	90.6 mm	155.4 mm	45.8 mm	80.1 mm
	Jun	103.7 mm	89.4 mm	126.8 mm	99.8 mm	230.6 mm	91.9 mm	47.6 mm
	Jul	105.1 mm	69.3 mm	252.6 mm	60.4 mm	123.3 mm	56.4 mm	69.9 mm
	Aug	77.7 mm	103.3 mm	233.2 mm	124.4 mm	146.9 mm	89.0 mm	96.5 mm
	Sep	22.2 mm	28.7 mm	37.0 mm	18.9 mm	35.1 mm	39.8 mm	74.0 mm
	Oct	13.9 mm	33.6 mm	23.5 mm	16.3 mm	11.8 mm	27.2 mm	51.0 mm
SG		Pseudo-gley	Haplic luvisol	Luvisol	Luvisol	Marsh	Cambisol	Haplic luvisol
ST		Loamy Sand	Silty clay	Sandy loam	Silty clay	Loamy sand	Clay loam	Loamy sand
SD		21.04.2021	25.05.2021	17.04.2021	30.04.2021	17.04.2021	05.05.2021	10.05.2021

**Table 2:** Means ($\bar{X}$), minimum ($X_{min}$), and maximum ($X_{max}$) of BLUPs, genotypic variance ($\sigma_{G}^{2}$), location variance ($\sigma_{L}^{2}$), genotype-by-location interaction variance ($\sigma_{GL}^{2}$), error variance ($\sigma_{\varepsilon }^{2}$), heritability ($h^{2}$) and repeatability ($w^{2}$), and $\sigma_{GL}^{2}$ to $\sigma_{G}^{2}$ ratio (σGL2σG2) across all environments, across environments within environmental target regions, and within single environments for seed yield (SY) in dt ha^−1^, protein content (PC) in %, oil content (OC) in %, plant height (PH) in cm, kernel dry matter (KDM) in %, and time between sowing and maturity (DTM) in days.

Trait	Location ^§^	$\bar{X}$	$X_{\min}$	$X_{\max}$	$\sigma_{G}^{2}$	$\sigma_{L}^{2}$	$\sigma_{GL}^{2}$	$\sigma_{\varepsilon }^{2}$	$h^{2}$	$w^{2}$	σGL2σG2
SY	Across	30.37	21.07	36.83	14.17 ***	17.78 ***	5.69 ***	9.60	0.90	-	0.40
	NTH	26.83	21.60	30.47	5.18 **	12.11 ^ns^	1.47 ^ns^	9.35	0.55	-	0.28
	STH	31.82	21.54	38.77	19.66 ***	14.69 ***	4.23 ***	10.01	0.91	-	0.22
	EWE	36.57	25.41	48.51	30.88 ***	-	-	13.63	-	0.82	
	HOH	26.74	18.00	30.82	13.91 ***	-	-	11.45	-	0.71	
	MOS	29.06	16.02	39.14	36.64 ***	-	-	6.60	-	0.92	
	NIT	33.81	18.07	41.72	21.47 ***	-	-	4.86	-	0.88	
	LDH	32.92	21.26	38.47	19.75 ***	-	-	8.80	-	0.79	
	BID	24.26	20.13	28.84	5.09 **	-	-	6.05	-	0.54	
	GÜL	29.35	23.67	33.16	9.45 *	-	-	12.58	-	0.53	
PC	Across	45.09	41.81	48.51	2.21 ***	4.45 ***	0.41 ***	0.73	0.94	-	0.19
	EWE	47.29	42.22	50.68	4.74 ***	-	-	0.51	-	0.94	
	HOH	47.22	43.35	50.42	2.72 ***	-	-	0.79	-	0.85	
	MOS	42.87	40.44	44.86	1.58 ***	-	-	0.34	-	0.89	
	NIT	44.69	40.39	49.01	3.50 ***	-	-	0.41	-	0.94	
	LDH	42.38	40.30	44.06	1.14 ***	-	-	0.39	-	0.82	
	BID	46.09	44.35	49.83	1.69 **	-	-	2.28	-	0.55	
OC	Across	17.47	16.48	18.7	0.31 ***	6.25 ***	0.16 ***	0.44	0.81	-	0.50
PH	Across	101.16	89.66	113.91	31.78 ***	75.48 ***	19.56 ***	46.15	0.79	-	0.62
KDM	Across	80.98	77.14	82.80	1.00 ***	3.36 ***	0.90 ***	0.98	0.82	-	0.90
DTM	Across	150.45	139.10	154.20	9.19 ***	88.41 ***	5.64 ***	3.36	0.87	-	0.62

^§^ NTH: BID and GÜL. STH: EWE, HOH, MOS, NIT and LDH. * *p* < 0.05, ** *p* < 0.01, *** *p* < 0.001, ^ns^ not significant.

**Table 3:** Shukla’s stability measure and mean ranks of 50 genotypes across two locations in the northern half (NTH) and five locations in the southern half (STH) of Germany. Mean SY represents BLUP values of seed yield in dt ha^−1^ across all locations within those environmental target regions for each genotype, ranking the highest value as 1 and the lowest value as 50. $\sigma_{i}^{2}$ represents Shukla’s stability variance, ranking the smallest, most stable value as rank 1 and the highest, least stable value as rank 50. Frames highlight the five highest-performing genotypes for both environmental target regions.

Genotype	Stability Rank STH	$\sigma_{i}^{2}$ STH	Rank SY STH	Mean SY STH	Stability Rank NTH ^§^	$\sigma_{i}^{2}$ NTH ^$^	Rank SY NTH	Mean SY NTH
G1	7	1.97	37	30.55	25	9.64	8	28.37
G2	42	15.18	31	31.56	26	10.56	22	27.17
G3	33	9.93	42	27.40	1	0	17	27.41
G4	43	15.67	48	22.95	17	4.95	42	24.07
G5	22	6.68	27	32.04	-	-	-	-
G6	38	12.86	35	30.69	11	2.45	35	25.26
G7	26	7.95	9	35.47	-	-	-	-
G8	16	4.81	2	38.04	1	0	20	27.29
G9	24	7.07	36	30.63	5	0.56	36	25.24
G10	36	11.88	38	30.25	20	6.91	9	28.19
G11	48	27.77	34	30.78	29	15.82	5	28.96
G12	5	1.75	21	33.69	12	2.57	3	29.19
G13	47	19.33	30	31.68	10	2.31	18	27.38
G14	28	8.50	22	33.38	18	4.98	21	27.20
G15	15	4.43	19	34.13	1	0	32	25.84
G16	44	15.94	40	29.27	-	-	-	-
G17	6	1.80	45	24.94	22	7.91	10	28.16
G18	13	3.70	28	32.00	23	8.32	28	26.28
G19	10	3.05	33	31.02	37	50.89	38	24.88
G20	21	5.85	15	34.82	33	23.15	12	28.07
G21	35	11.16	16	34.33	-	-	-	-
G22	14	3.95	24	32.97	6	0.61	13	28.06
G23	17	4.85	26	32.12	15	3.33	37	25.20
G24	9	3.05	12	35.11	1	0	11	28.08
G25	18	5.42	7	36.15	1	0	7	28.48
G26	8	2.18	13	34.94	11	2.45	31	25.97
G27	4	1.23	4	36.86	21	7.74	26	26.68
G28	23	6.91	20	34.03	24	8.75	27	26.40
G29	39	13.27	8	35.58	7	0.76	6	28.59
G30	2	0.85	18	34.18	35	37.47	33	25.80
G31	25	7.87	29	31.71	34	33.10	24	26.99
G32	49	30.04	3	36.99	16	4.66	14	28.06
G33	45	16.16	25	32.48	27	13.64	39	24.81
G34	30	0.85	17	34.31	2	0.14	29	26.20
G35	41	7.87	1	38.77	13	2.69	1	30.47
G36	3	30.04	32	31.25	4	0.32	15	27.92
G37	11	3.11	5	36.35	14	3.18	2	29.90
G38	34	10.24	46	24.61	19	5.46	41	24.51
G39	40	13.51	50	21.54	3	0.27	43	23.71
G40	1	0.40	44	25.41	32	21.10	30	26.06
G41	19	5.56	49	22.14	30	16.16	44	21.60
G42	20	5.68	10	35.33	-	-	-	-
G43	27	8.07	11	35.15	9	1.67	4	29.02
G44	50	38.57	47	24.16	38	83.21	40	24.70
G45	32	9.76	14	34.84	28	15.53	19	27.35
G46	37	12.84	41	28.56	1	0	23	27.14
G47	31	9.75	39	29.93	31	17.29	34	25.57
G48	29	8.61	6	36.20	8	1.59	25	26.85
G49	46	17.5	23	33.22	36	47.85	16	27.78
G50	12	3.23	43	26.52	-	-	-	-

^§^ Missing values were due to missing observations in GÜL. ^$^ Negative estimated values were set to 0.

## Data Availability

Data is contained in the Supplementary Materials.

## Supplementary Materials

The following supporting information can be downloaded at: https://www.mdpi.com/article/10.3390/plants12040756/s1, Figure S1: Map of (a) locations and plots of (b) daily mean temperature and (c) precipitation summed up for each week from sowing to harvest for each location. Colours of locations in (a) indicate the locations in plots (b) and (c). Crosses in (a) show locations that could not be harvested; Figure S2: Correlation matrix for seed yield between seven locations; Figure S3: Correlation matrix for protein content between six locations; Figure S4: Correlation matrix for six traits across all locations; Table S1: Shukla’s stability measure and mean ranks of 50 genotypes for seed yield across seven locations; Table S2: Shukla’s stability measure and mean ranks of 50 genotypes for protein content across six locations; Table S3: List of genotypes evaluated in this study; data and data information.
