# Supplementary material for: Do We Need to Breed for Regional Adaptation in Soybean?—Evaluation of Genotype-by-Location Interaction and Trait Stability of Soybean in Germany
Source: Plants (Basel). 2023 Feb 8;12(4):756. doi: 10.3390/plants12040756 (PMC9959684; doi:10.3390/plants12040756)
Supplement: Supplementary file 1 [file plants-12-00756-s001.zip › Supplementary Material Revised.pdf]

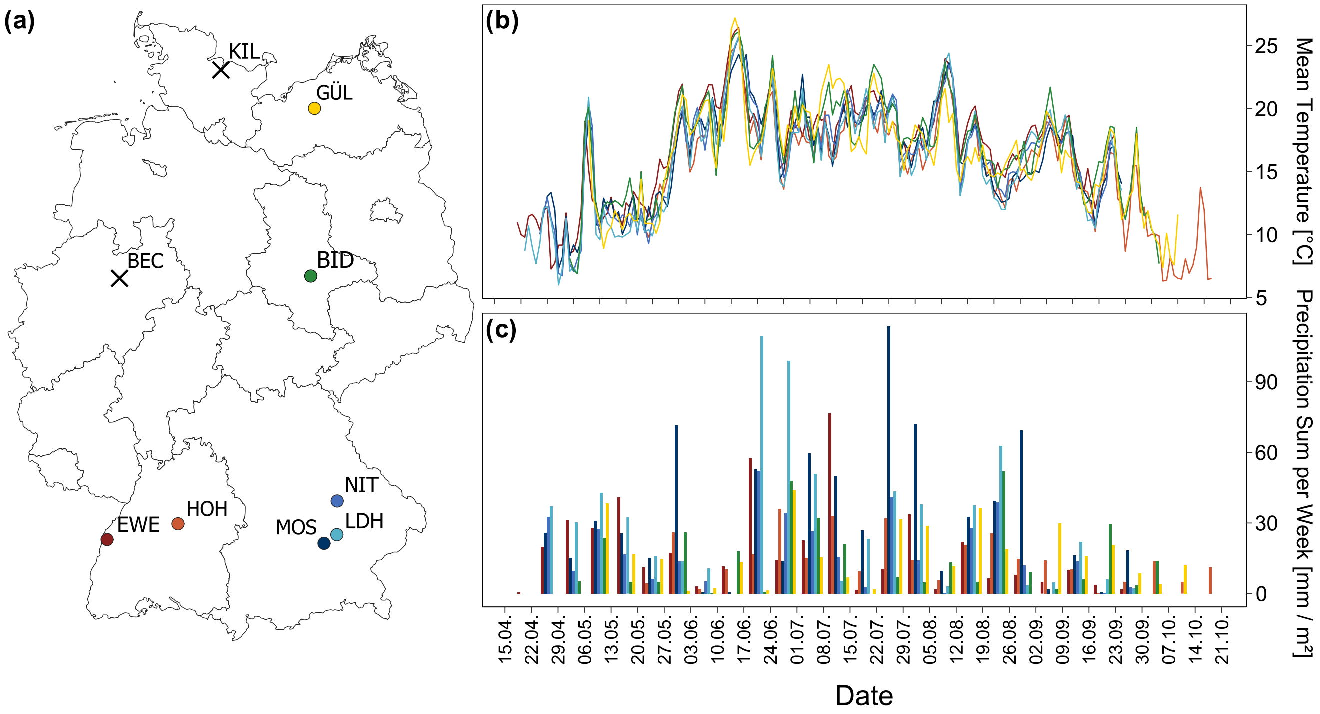

Figure S1. Map of (**a**) locations and plots of (**b**) daily mean temperature and (**c**) precipitation summed up for each week from sowing to harvest for each location. Colours of locations in (**a**) indicate the locations in plots (**b**) and (**c**). Crosses in (**a**) show locations that could not be harvested.

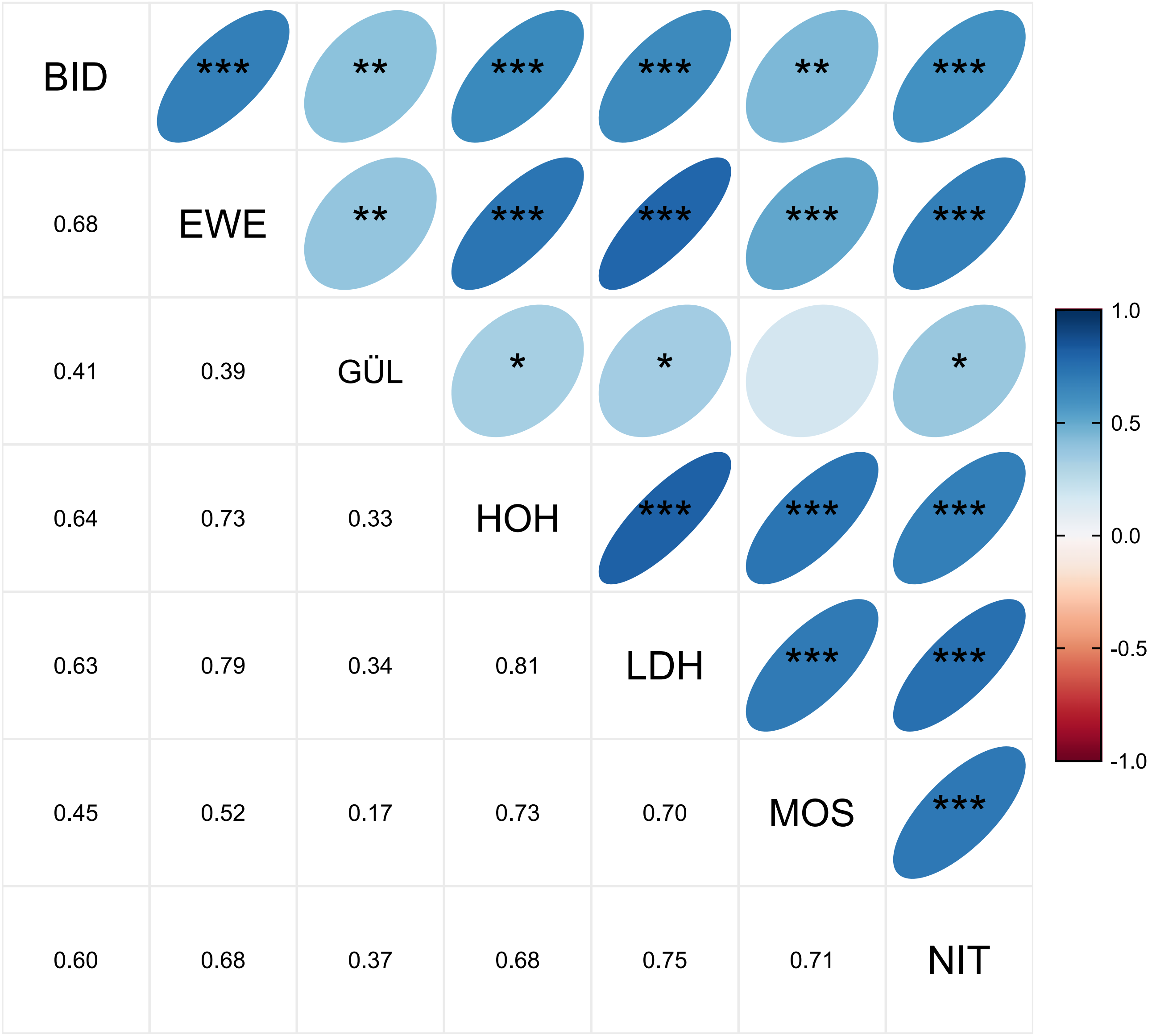

Figure S2. Correlation matrix for seed yield between seven locations.
*P < 0.05, **P < 0.01, ***P < 0.001

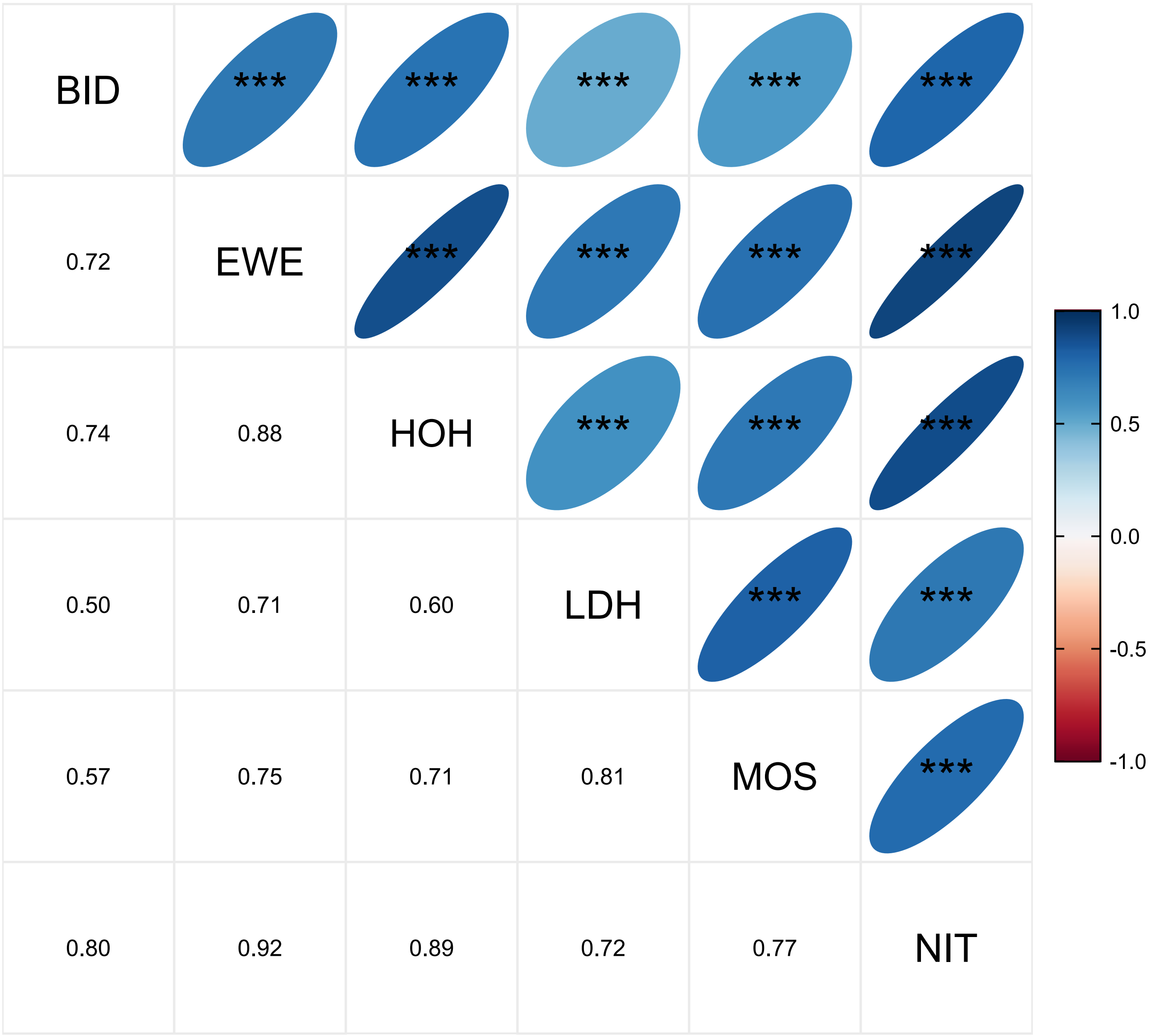

Figure S3. Correlation matrix for protein content between six locations.
***P < 0.001

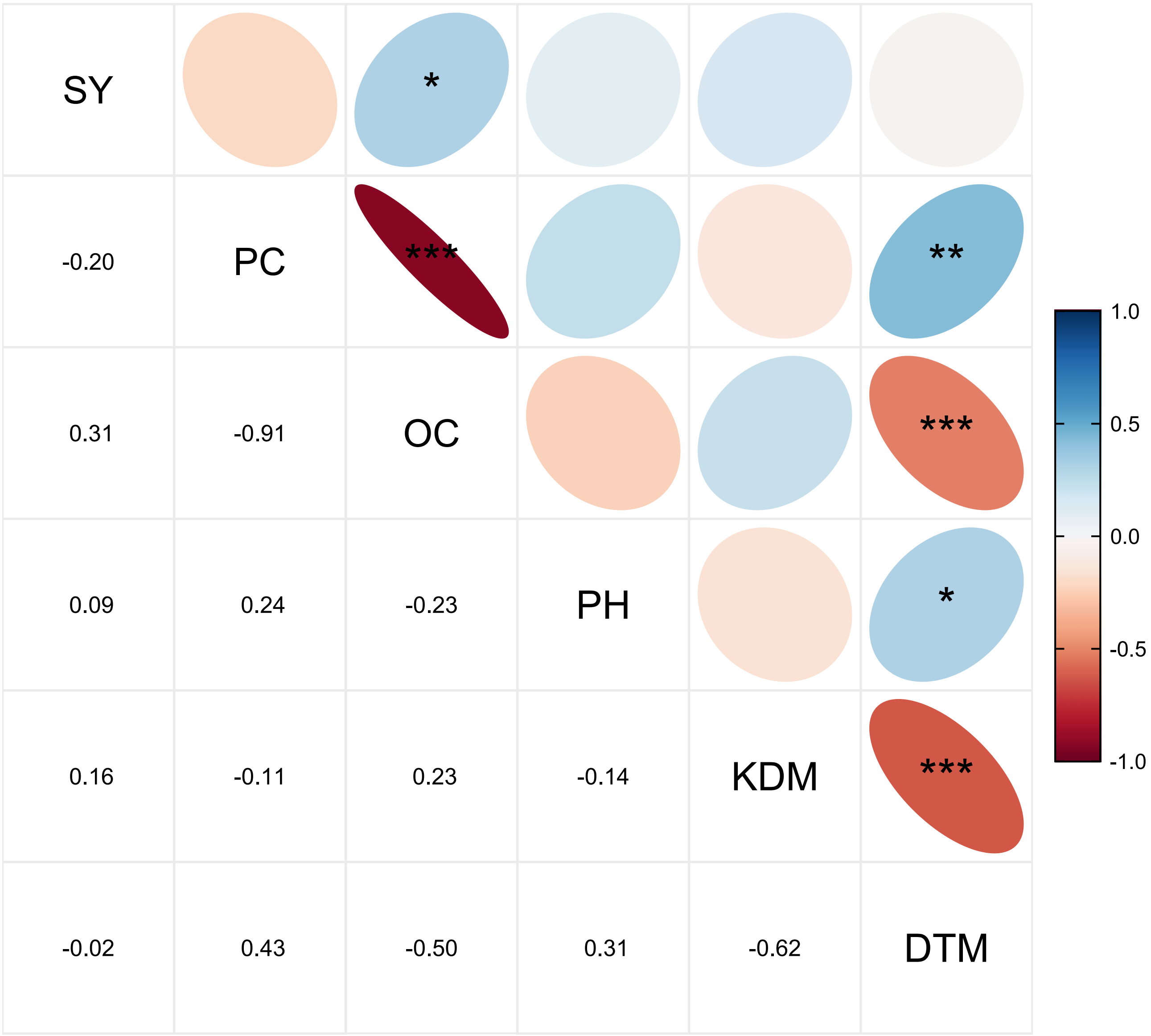

Figure S4. Correlation matrix for six traits across all locations. The traits are seed yield (SY), protein content (PC), oil content (OC), plant height (PH), kernel dry matter content (KDM), and days to maturity (DTM). *P < 0.05, **P < 0.01, ***P < 0.001

Table S1. Shukla's stability measure and mean ranks of 50 genotypes for seed yield (SY) across seven locations. Mean SY represents BLUP values across all locations for each genotype, ranking the highest value as 1 and the lowest value as 50. σ_i^2 represents Shukla's stability variance, ranking the smallest, most stable value as rank 1 and the highest, least stable value as rank 50.

Genotype	Rank Shukla	σ_i^2	Rank Mean SY	Mean SY[§]
G1	12	6.07	33	$30.07^{efghijk}$
G2	33	12.05	30	$30.36^{efghijkl}$
G3	42	15.76	42	27.52^{cde}
G4	45	19.39	48	22.76^{ab}
G5	9	4.76	25	$30.61^{efghijklmn}$
G6	21	9.19	37	28.87^{defghi}
G7	13	6.08	9	33.51^{lmnopq}
G8	34	12.51	2	34.91^{pq}
G9	10	5.76	38	28.81^{defgh}
G10	35	12.80	35	29.83^{efghij}
G11	49	25.84	26	$30.53^{efghijklmn}$
G12	3	2.84	14	$32.78^{jklmnop}$
G13	37	13.47	27	$30.51^{efghijklm}$
G14	4	2.87	22	$31.70^{ghijklmnop}$
G15	16	7.23	23	$31.60^{fghijklmnop}$
G16	41	14.41	39	28.61^{cdefgh}
G17	48	24.53	43	25.87^{bcd}
G18	8	4.74	31	$30.25^{efghijkl}$
G19	39	13.65	36	28.97^{defghi}
G20	22	9.28	12	$32.99^{jklmnop}$
G21	18	7.71	16	$32.52^{jklmnop}$
G22	15	7.16	21	$31.71^{ghijklmnop}$
G23	19	8.07	34	29.92^{efghij}
G24	1	2.27	11	33.27^{klmnop}
G25	7	4.07	5	34.19^{opq}
G26	17	7.44	17	$32.18^{ijklmnop}$
G27	25	9.94	7	33.85^{nopq}
G28	27	10.45	19	$31.80^{ghijklmnop}$
G29	20	9.05	6	33.96^{opq}
G30	31	11.70	24	$31.56^{fghijklmno}$
G31	32	11.79	28	$30.47^{efghijkl}$
G32	47	23.47	4	34.59^{opq}
G33	44	18.39	32	$30.11^{efghijk}$
G34	24	9.50	18	$31.92^{hijklmnop}$
G35	28	10.83	1	36.83^{q}
G36	5	3.06	29	$30.43^{efghijkl}$
G37	2	2.54	3	34.89^{opq}
G38	30	11.67	46	24.13^{ab}
G39	43	18.28	49	21.57^{a}
G40	40	14.33	45	25.42^{bc}
G41	14	6.42	50	21.07^{a}
G42	38	13.50	15	$32.68^{jklmnop}$
G43	11	5.82	8	33.83^{mnopq}
G44	50	42.48	47	23.79^{ab}
G45	26	10.32	13	$32.97^{jklmnop}$
G46	36	13.21	41	28.28^{cdef}

G47	23	9.33	40	28.56[cdefg]
G48	29	11.12	10	33.49[lmnopq]
G49	46	19.9	20	31.75[ghijklmnop]
G50	6	3.40	44	25.64[bcd]

[§]Mean values assigned with the same letter are not significantly different from each other.
LSD (α = 0.05) = 3.34

Table S2. Shukla's stability measure and mean ranks of 50 genotypes for protein content (PC) across six locations. Mean PC represents BLUP values of protein content across all locations for each genotype, ranking the highest value as 1 and the lowest value as 50. σ_i^2 represents Shukla's stability variance ranking the smallest, most stable value as rank 1 and the highest, least stable value as rank 50.

Genotype	Rank Shukla	σ_i^2	Rank Mean PC	Mean PC
G1	29	0.50	37	44.28
G2	43	1.37	49	41.93
G3	17	0.33	15	45.92
G4	9	0.22	8	46.55
G5	31	0.57	20	45.66
G6	10	0.24	30	45.27
G7	26	0.49	35	44.36
G8	19	0.34	34	44.48
G9	18	0.34	18	45.85
G10	33	0.66	9	46.36
G11	35	0.77	29	45.37
G12	3	0.10	19	45.74
G13	22	0.37	4	46.64
G14	20	0.35	24	45.53
G15	42	1.33	26	45.50
G16	16	0.32	5	46.60
G17	36	0.79	6	46.59
G18	15	0.32	7	46.57
G19	38	0.98	17	45.89
G20	41	1.30	38	44.14
G21	13	0.29	23	45.53
G22	30	0.57	32	44.87
G23	12	0.26	40	43.92
G24	21	0.36	10	46.30
G25	6	0.16	27	45.48
G26	8	0.18	28	45.45
G27	11	0.26	41	43.89
G28	44	1.37	31	45.16
G29	2	0.05	12	46.14
G30	4	0.10	21	45.60
G31	7	0.18	22	45.54
G32	23	0.38	42	43.88
G33	24	0.39	2	46.74
G34	25	0.49	45	43.14
G35	5	0.13	33	44.56
G36	32	0.64	14	45.95

G37	34	0.74	3	46.72
G38	49	2.41	1	48.51
G39	28	0.50	25	45.50
G40	14	0.31	11	46.26
G41	37	0.95	13	46.06
G42	39	1.08	16	45.90
G43	1	0.03	39	43.98
G44	45	1.40	46	42.80
G45	48	2.40	47	42.22
G46	46	1.62	43	43.57
G47	40	1.14	50	41.81
G48	27	0.50	36	44.35
G49	47	1.84	48	42.04
G50	50	6.01	44	43.35

Table S3. List of genotypes evaluated in this study.

Genotype	Genotype Name	Pedigree	Maturity Group
G1	Tofina	Tofina	000
G2	Merlin	Merlin	000
G3	Lenka	Lenka	00
G4	Simocine SZS	Simocine SZS	000
G5	SJE17-2708-n-n-n-4867-008	Lenka × EWE 20	-
G6	SJE15-418-02-03-n-102-n	EWE 0013 × EWE 0004	-
G7	SJE17-2716-n-n-n-5820-001	ES Mentor × (Protina × Aveline)	-
G8	SJE17-2718-n-n-n-6093-034	ES Mentor × EWE 20	-
G9	SJE17-2708-n-n-n-4740-001	Lenka × EWE 20	-
G10	SJE17-2708-n-n-n-4743-004	Lenka × EWE 20	-
G11	SJE17-2708-n-n-n-4745-006	Lenka × EWE 20	-
G12	SJE17-2708-n-n-n-4749-010	Lenka × EWE 20	-
G13	SJE17-2708-n-n-n-4750-011	Lenka × EWE 20	-
G14	SJE17-2708-n-n-n-4754-015	Lenka × EWE 20	-
G15	SJE17-2708-n-n-n-4862-003	Lenka × EWE 20	-
G16	SJE17-2708-n-n-n-4871-012	Lenka × EWE 20	-
G17	SJE16-2020-n-n-n-1088-001	Lenka × (Silvia × EWE 0016)	-
G18	SJE16-2004-n-n-n-0121-001	Lenka × Sigalia	-
G19	SJE16-2002-n-n-n-0002-002	Lenka × Tofina	-
G20	SJE16-2002-n-n-n-0003-003	Lenka × Tofina	-
G21	SJE16-2002-n-n-n-0004-004	Lenka × Tofina	-
G22	SJE16-2002-n-n-n-0009-009	Lenka × Tofina	-
G23	SJE16-2002-n-n-n-0017-017	Lenka × Tofina	-
G24	SJE17-2723-n-n-n-6486-006	Amadea × EWE 20	-
G25	SJE17-2718-n-n-n-6060-001	ES Mentor × EWE 20	-
G26	SJE17-2718-n-n-n-6061-002	ES Mentor × EWE 20	-
G27	SJE17-2718-n-n-n-6069-010	ES Mentor × EWE 20	-
G28	SJE17-2718-n-n-n-6083-024	ES Mentor x EWE 20	-
G29	SJE17-2718-n-n-n-6084-025	ES Mentor × EWE 20	-

G30	SJE17-2718-n-n-n-6091-032	ES Mentor × EWE 20	-
G31	SJE17-2718-n-n-n-6096-037	ES Mentor × EWE 20	-
G32	SJE17-2718-n-n-n-6098-039	ES Mentor × EWE 20	-
G33	SJE17-2716-n-n-n-5821-002	ES Mentor × (Protina × Aveline)	-
G34	SJE16-2069-n-n-n-2475-003	ES Mentor × Lenka	-
G35	SJE16-2069-n-n-n-2476-004	ES Mentor × Lenka	-
G36	SJE17-2713-n-n-n-5340-001	ES Commander × EWE 20	-
G37	SJE17-2713-n-n-n-5464-005	ES Commander × EWE 20	-
G38	SJE13-047-n-n-p-001-110-n	(Primus × Sultana) × Protibus	-
G39	SJE13-047-n-n-p-001-111-n-n	(Primus × Sultana) × Protibus	-
G40	SJE13-047-n-n-p-001-121-n	(Primus × Sultana) × Protibus	-
G41	SJE13-047-n-n-p-001-210-n	(Primus × Sultana) × Protibus	-
G42	SJE17-2718-n-n-n-6100-041	ES Mentor × EWE 20	-
G43	SJE17-2723-n-n-n-6483-003	Amadea × EWE 20	-
G44	Smaragd	Smaragd	0
G45	Nessie PZO	Nessie PZO	000
G46	Magnolia PZO	Magnolia PZO	000
G47	Adessa	Adessa	0000
G48	Achillea	Achillea	000
G49	Wapiti	Wapiti	000
G50	Solena	Solena	000/00
